# Medical Society of London

**Published:** 1883-05

**Authors:** 


### MEDICAL SOCIETY OF LONDON.

Sir Joseph Fayrer, K.C.S.I., M D., President, in the Chair.

*Poisoning by Citrate of Caffeine.*—Dr. Routh read a paper on a case of poisoning by citrate of caffeine. The effervescent citrate of Bishop was intended, but the pure citrate was sent. The patient recovered. The President did not know of any similar case in men. The caffeine was like theine and the alkaloid of cocoa and Paraguay tea, in increasing the power of muscular effort. Dr. Thorowood mentioned cases in which citrate of caffeine had been useful in cases of asthma. Dr. Gilbart Smith had given citrate of caffeine in cardiac and renal disease, and in asthma. Dr. Routh, in reply, had noticed no convulsions, only marked muscular tremors. There was certainly paralysis, and the man had to be carried up-stairs. There was nothing like tetanus.

*The Antiseptic Treatment of Pulmonary Disease.*—Dr. Lee remarked that the principles on which antiseptic surgery were founded, might be extended to the treatment of pulmonary diseases, provided that the difference between diffusion in a vapour and a fluid were attended to. In the volatilization of any antiseptic or medicinal agent, it was necessary that the water with which it was mixed should be evaporated; and no practical use resulted from simply mixing the substance with hot water, and breathing the steam. Dr. Lee stated that the rate of volatilization of any substance when boiled with water depended on its own boiling point, its specific gravity, and its readiness to mix with water. In the case of the oil of the eucalyptus globulus, by mixing alcohol with it, the rate of evaporation could be controlled; for, though its boiling point was 320°, its specific gravity was less than water, and it volatilized when mixed with it much more readily than the water. Carbolic acid had the singular property of volatilizing in exactly the same proportion as the water with which it was mixed; and thus it was the most suitable for all antiseptic methods of treatment. Dr. Cullimore, Dr. Drewett, and the President, made remarks.

BORDER COUNTIES BRANCH.

*Tracheotomy.*—Dr. J. A. Macdougall (Carlisle) read a paper on this subject. The primary object with which the note was read was to draw attention to the fact that the trachea, especially in young children, could with perfect safety be opened through the isthmus of the thyroid; and that, this being the case, the high operation was the one which, in ordinary circumstances, should be selected. It was also urged that, in severe cases of true croup, if decided amendment did not follow active treatment, carefully carried out for eight hours, the best chance of recovery lay, in the majority of instances, in tracheotomy; further, that, the existing condition having become so urgent as to demand consideration of the operation, its performance was not to be delayed on account of apparent amelioration—an amelioration of which time too surely proved the falseness.

*Case of Pelvic Abscess.*—Dr. Muir of Selkirk described this case. The patient, a man aged 38, who had never been ill before, was seized with perityphilitis, which ended in suppuration. The abscess seemed to be pointing above the groin, but the tumor suddenly disappeared, and coincidently there were symptoms of intestinal obstruction and retention of urine, accompanied by alarming collapse, tympanitis, hiccough, and vomiting; all of which were completely and rapidly relieved on the evacuation of a quantity of fœtid pus by means of an aspirator introduced

*per rectum,* and puncturing a fluctuating tumor felt there. A week afterwards, the dangerous symptoms returned, and were again relieved in the same way. Complete recovery followed.

*The Treatment of Enlarged Glands in the Neck.*—Dr. Lediard drew attention to the frequency of this disease in Cumberland, and the absence of any specific remedy. Brief allusion was made to the pathology of strumous glands, and the subsequent changes of caseation and suppuration, these conditions being the ones most suitable for operative measures, which were recommended in chronic cases where there was no sign of yielding to other treatment. Billroth's operations were adduced in support of the paper, as well as Treves's rule for interference. Extirpation was to be made through skin-incisions near the sterno-mastoid muscle; the glands teased out if firm, but scooped out of their capsules if soft and adherent. Dr. Lediard's own experience was limited to five cases, in all of which the results had been satisfactory.

